# Supplementary material for: Empirical methods for controlling false positives and estimating confidence in ChIP-Seq peaks
Source: BMC Bioinformatics. 2008 Dec 5;9:523. doi: 10.1186/1471-2105-9-523 (PMC2628906; doi:10.1186/1471-2105-9-523)
Supplement: Additional File 1 — A variety of html documents from the USeq web site detailing the available applications, their best usage, output file type descriptions, command line menus, etc. [file 1471-2105-9-523-S1.zip › USeqUserGuides/JavaDoc.zip!/JavaDoc/edu/utah/seq/methylation454/MethylationParser.html]

MethylationParser


|  |  |  |
| --- | --- | --- |
| **Overview** \| **Package** \| **Class** \| **Use** \| **Tree** \| **Deprecated** \| **Index** \| **Help** | |  |
| **PREV CLASS**   **NEXT CLASS** | **FRAMES**    **NO FRAMES**     **All Classes** |  |
| SUMMARY: NESTED \| FIELD \| CONSTR \| METHOD | DETAIL: FIELD \| CONSTR \| METHOD |  |


---


## edu.utah.seq.methylation454 Class MethylationParser

```
java.lang.Object
  edu.utah.seq.methylation454.MethylationParser
```

---

``` public class MethylationParser extends java.lang.Object ```

---

**Constructor Summary**

`MethylationParser(java.io.File dataFile)`


| **Method Summary** | |
| --- | --- |
| `void` | `closeSetAmplicons()` |
| `void` | `closeSetCpGs()` |
| `void` | `closeSetReads()` |
| `Sample[]` | `getSamples()` |
| `void` | `makeSamples()` |
| `void` | `parseDataLines(java.io.File dataFile)` |
| `void` | `parseIt(java.io.File dataFile)` |

**Methods inherited from class java.lang.Object**

`equals, getClass, hashCode, notify, notifyAll, toString, wait, wait, wait`

**Constructor Detail**

### MethylationParser

```
public MethylationParser(java.io.File dataFile)
```


**Method Detail**

### parseIt

```
public void parseIt(java.io.File dataFile)
```

---


### makeSamples

```
public void makeSamples()
```

---


### closeSetAmplicons

```
public void closeSetAmplicons()
```

---


### closeSetCpGs

```
public void closeSetCpGs()
```

---


### closeSetReads

```
public void closeSetReads()
```

---


### parseDataLines

```
public void parseDataLines(java.io.File dataFile)
```

---


### getSamples

```
public Sample[] getSamples()
```


---


|  |  |  |
| --- | --- | --- |
| **Overview** \| **Package** \| **Class** \| **Use** \| **Tree** \| **Deprecated** \| **Index** \| **Help** | |  |
| **PREV CLASS**   **NEXT CLASS** | **FRAMES**    **NO FRAMES**     **All Classes** |  |
| SUMMARY: NESTED \| FIELD \| CONSTR \| METHOD | DETAIL: FIELD \| CONSTR \| METHOD |  |


---
